# On Arsenical Paper-Hangings

**Published:** 1859-06

**Authors:** Alfred S. Taylor


### ECLECTIC DEPARTMENT,

### AND SPIRIT OF THE MEDICAL PERIODICAL PRESS.

---

*On Arsenical Paper-Hangings. The mode in which they may produce noxious effects.* By ALFRED S. TAYLOR, M. D.

A friend, whose library walls were covered with an arsenical-green paper, had suffered for some time from chronic inflammation of the eyes, especially affecting the conjunctivæ of the eyelids. On the discovery that arsenic was contained in the green pigment of this paper in rather large quantity, he caused it to be removed during the summer and replaced by another containing no arsenic. The inflammation from which he had suffered disappeared; but within the last few weeks it has returned. He informed me that he had been dusting some books in a book-case belonging to this room, and he imagined that the dust, which had accumulated for two or three years, had affected his eyes, and had caused a return of the inflammation. Some of the dust was carefully removed on Tuesday last from the top of a few books by a feather, and submitted to a chemical analysis. The dust weighed one grain and a half; it had an olive-green color, and under the microscope it presented the appearance of fibres, with numerous particles of various colors, chiefly of a greyish black. By Reinsch's process, a portion of the dust yielded a deposit of arsenic, and there was, therefore, clear evidence that some of the mineral arsenical pigment, formerly on the walls, had found its way through the glass doors of the bookcase, and had been deposited in the form of a fine dust on the tops of the books.

On Thursday last, after having made this chemical examination of the dust from a private dwelling, I procured from the shop of Messrs. Marratt & Short, opticians, 63 King William Street, London-bridge, a quantity of dust for the purposes of analysis. The walls of this shop are covered with an unglazed arsenical paper; and, as I am informed, they have been so covered for a period of about three years. In collecting this dust from the tops of the cases containing the instruments, great care was taken not to touch the walls. The quanity thus collected for examination amounted to about four hundred and fifty grains. It was nearly black, and under the microscope appeared to consist of fibres and sooty particles. It was very light and flocculent. One hundred and fifty grains of this dust were examined by Reinsch's process, and enough metallic arsenic was obtained from it to coat about ten square inches of copper foil, in addition to a piece of copper gauze. From the latter deposit, by the application of heat, octahedral crystals of arsenic were readily obtained. The cases had not been dusted for a period of nine months.

The cases are secured by glass doors, and lined inside at the back with arsenical paper. Mr. Short removed, by a camel's hair pencil, a small quantity of dust which had accumulated on the thermometers and barometers, which are locked up within the closet. The quantity thus obtained from the projecting parts of a few instruments, amounted to eight-tenths of a grain, of which five-tenths were taken for examination. This half grain of dust sufficed to cover pretty thickly with metallic arsenic a square inch of copper gauze. A portion of this, when heated, yielded a large number of well-defined octahedral crystals of arsenious acid.

These facts lead to the inevitable inference that the air of a room, of which the walls are covered with an unglazed arsenical-green paper, is liable to be charged with the fine dust of the poisonous aceto-arsenite of copper. Those who inhabit these rooms are exposed to breathe the dust. The poison may thus find its way by the pulmonary membrane into the system, or it may affect the eyes, nose and throat by local action. That so few cases of actual poisoning, under these circumstances, have occurred is a fortunate circumstance; but only cases of serious mischief would be likely to attract attention. There may have been numerous instances of a disturbance of health, depending on this arsenical paper, which from absence of suspicion has been assigned to other causes. The degree of exposure, state of health, peculiar susceptibility, and the eliminating power of the system, may account for the comparative rareness of these cases. The mode in which the pigment is laid on the paper may be such as to prevent, in some instances, the fine particles of the dust from escaping. The fact now demonstrated, that arsenical dust is, and must be, substantially breathed by those who occupy rooms thus papered, explains the similarity of symptoms observed; justifies the statements made by Dr. Hinds, Dr. Halley, and others, and proves that those who have experimented on this subject with negative results, have not taken the right course to arrive at the truth. Their results have, to a certain extent, misled the public, by teaching them to rely on what is now proved to be a false security.

If, as a general rule, the quantity of arsenic which can penetrate the body from this source is small, it is still desirable that arsenic should not be breathed day by day in any proportion. The defenders of this noxious manufacture will hardly go the length of asserting that this arsenical-green, which is a potent poison in the stomach, can exert no injurious effect when taken into the lungs; and yet, unless this assumption be made, the inevitable inference is, that these papers should not be used for covering the walls of our dwellings.—*Med. Times and Gaz.*, Jan 1, 1859.

